# Prevalence of Screening for Alcohol Use by Physicians During Routine Physical Examinations

**Published:** 1994

**Authors:** Diane Deitz, Fred Rohde, Darryl Bertolucci, Mary Dufour

**Affiliations:** Diane Deitz, Ph.D., and Fred Rohde, M.A., are research associates at Cygnus Corporation, Washington, DC, and analysts for the National Institute on Alcohol Abuse and Alcoholism’s (NIAAA) Alcohol Epidemiologic Data System (AEDS). Darryl Bertolucci, M.A., is a mathematical statistician for and the acting deputy director of the Epidemiology Branch, Division of Biometry and Epidemiology, NIAAA, Rockville, Maryland. Mary Dufour, M.D., M.P.H., is acting deputy director of NIAAA, Rockville, Maryland

## Abstract

In the survey presented here, primary care physicians were asked whether physicians inquired about their alcohol consumption during routine office visits. Results show that both demographics and the patient’s health status affect how often the physician asked about alcohol use. In addition, the majority of survey respondents, who were seen for a checkup within the last 2 years, were not asked about their alcohol consumption.

This Epidemiologic Bulletin examines the screening of patients for alcohol consumption by physicians during routine office visits. Screening is a medical procedure designed to identify a treatable disease, preferably in its early or presymptomatic stage. Respondents’ reports of screening for alcohol consumption by their physicians during an office visit are compared with their reported typical alcohol consumption patterns and with selected demographic characteristics. Data for our analysis were extracted from the 1991 National Health Interview Survey (NHIS) of Health Promotion and Disease Prevention (HPDP).

The NHIS is an ongoing, nationally representative, household interview survey of the population, conducted by trained interviewers for the National Center for Health Statistics (NCHS). Interviewers travel to eligible households and conduct face-to-face interviews with all available family members. Of the 48,853 households eligible for interview in 1991, 46,761 households were interviewed, resulting in a sample of 120,032 people. The HPDP contains questions on basic health habits, including alcohol use, as well as questions on disease prevention knowledge and practices. The HPDP sample consists of adults age 18 and older, randomly selected from the NHIS sample. One adult per family is selected to answer questions on the HPDP portion of the NHIS.

## Background

Alcohol abuse and alcoholism are major public health concerns. The misuse of alcohol contributes to an array of health and social problems, including, but not limited to, liver and neurologic disease, accidents, birth defects, disability, and homelessness. The economic costs of these problems to the Nation are staggering. When direct costs (i.e., treatment and support) and indirect costs (e.g., productivity loss and property damage) are combined, the resulting costs are estimated to be in the billions (Rice 1993).

The adverse health consequences of alcohol consumption have been documented extensively (National Center for Health Statistics 1992). It has been estimated that 20 percent or more of all general hospital admissions are alcohol related (Lewis and Gordon 1983) and that in the ambulatory care sector, approximately 15 percent of all office visits to a physician may be related to problems with alcohol (Hingson et al. 1982). The amount of alcohol consumed is closely related to health outcomes, and a 1988 study by Babor and colleagues indicated that there is generally a direct relationship between the level of alcohol consumed and the extent of problems experienced.

Despite the documented relationship between health problems and alcohol, many people will seek help from their physicians without readily acknowledging, or being aware of, the critical role that alcohol consumption may be playing in the etiology of their complaints. Consequently, the responsibility for identification, and subsequent treatment of alcohol-related problems, rests within the physicians’ domain.

The importance of screening for alcohol problems in general medical practice has been stressed by medical professionals (Bowen and Sammons 1988). The office visit provides a valuable opportunity for the physician to discuss general health habits, including alcohol consumption, with the patient, and the clinical setting is an effective and appropriate place for the delivery of preventive services, including screening and counseling. The overall effectiveness of preventive services in reducing disease, disability, and premature death is well documented (U.S. Department of Health and Human Services [USDHHS] 1991).

Accordingly, national support for the prevention of health problems through screening, education, and early detection and intervention is demonstrated in the agenda for *Healthy People 2000*. This program’s purpose is to decrease preventable diseases and deaths via three broad categories of activities: health promotion, health protection, and preventive services (USDHHS 1991). One goal of this agenda is to reduce alcohol and other drug abuse by reducing aggregate per capita alcohol consumption nationally, increasing public awareness of the harmful effects of addictive substances, improving access to treatment programs, and stimulating primary care providers to become more involved with these problems (USDHHS 1991).

A first step toward increasing the involvement of primary care providers is to focus attention on their assessment of whether patients consume alcohol and their determination of its potential impact on their patients’ health. Researchers exploring this issue must consider how often physicians inquire about alcohol consumption during patient visits to their offices. The purpose of our analysis is threefold: (1) to examine the percentage of women and men age 18 and older who report that they were asked about their alcohol consumption during visits to their private physician; (2) to examine whether results of screening differ among demographic subgroups within the population studied (e.g., women of childbearing age); and (3) to study whether results of screening differ among respondents who reported abstinence, current drinking, and various levels of drinking to questions in the HPDP. It is important to remember that the results are based to some extent on patient recall, which may be of questionable accuracy.

## Methods

### Clinic Visit Classifications

We used the clinical and preventive services sections of the HPDP in our analysis to determine the extent of physician screening for alcohol use and other potentially detrimental health practices. Respondents answering the HPDP had been asked to describe any physician consultations that involved examination, diagnosis, treatment, or advice. Respondents also had been asked to describe activities associated with this consultation, including whether physician screening for smoking, drinking, and other drug use occurred. The estimation of alcohol consumption is based on the respondents’ reported consumption for the 2 weeks prior to the NHIS interview.

All respondents who claimed to have received clinical services (defined as a visit to a health care practitioner) within 2 years prior to the HPDP interview were included in the analysis. Although the primary focus of this analysis is on screening for alcohol consumption, we included screening for smoking and other drug use for comparison with alcohol screening. It should be noted that alcohol consumption levels were obtained at the time of the HPDP interview and not at the time of the clinic visit. Consequently, the level of alcohol consumption reported on the interview may vary from consumption reported during the time of the checkup.

Questions from the clinical and preventive services section of the HPDP included the following: How long has it been since your last routine checkup by a medical doctor or other health professional? What kind of place was it: a clinic, a health center, a hospital, a doctor’s office, or some other place? During this last checkup were you asked how much and how often you drink alcohol? During this last checkup were you asked whether you smoke cigarettes or use other forms of tobacco? During this last checkup were you asked whether you use marijuana, cocaine, or other drugs?

### Alcohol Use Classifications

We derived levels of alcohol consumption from three items on the alcohol section of the HPDP. Questions from the alcohol section included the following: Have you had at least one drink of beer, wine, or liquor during the past year? During the past 2 weeks, on how many days did you drink any alcoholic beverages, such as beer, wine, or liquor? On the day(s) that you drank alcoholic beverages, how many drinks did you have (per day on the average)?

The first question asked whether respondents had consumed at least one drink during the past year. Respondents answering no to this question were classified as “abstainers” by the interviewer and were not required to answer any further alcohol questions. Respondents answering yes to this question were asked additional questions that provided information on the frequency and quantity of alcohol consumed.

We derived the quantity/frequency (QF) measure by multiplying the average number of drinks per day by the number of days alcohol was consumed in the past 2 weeks. The drinking levels obtained on the QF measure were used to construct drinking classifications for nonabstainers (i.e., infrequent, light, moderate, and heavy). These classifications differ from alcohol classifications of previous studies using the HPDP.^1^ In our analysis, the drinking classification “infrequent” refers to respondents who drank within the last year but did not drink during the 2 weeks prior to their interview. “Light” refers to respondents who drank one to seven drinks within the prior 2 weeks. “Moderate” refers to respondents who drank 8 to 26 drinks during the prior 2 weeks. “Heavy” refers to respondents who drank 27 or more drinks during the prior 2 weeks.

## Analysis and Limitations of the Data

Basic characteristics of the study sample are presented as unweighted numbers^2^ and weighted percentages.^3^ Percentages are used in all comparison analyses and are generated from weighted sample data. Weighted sample calculations are provided by NCHS with the data to allow estimations of national distribution. Respondents’ reported screening for alcohol consumption during a checkup are compared using demographic and personal characteristics as well as drinking behaviors. A “worst case” sampling design effect also was incorporated into the analysis.^4^ The tables presented reflect weighted data and where applicable, the sample size used in the calculation of the estimate.

Multiple logistic regression analysis was conducted next to control for confounding and to identify the variables most predictive of screening for alcohol consumption. The variables entered into the model were sex, race, age, education, poverty status, health status, place of screening, marital status, smoking status, and the five levels of alcohol consumption. Age and education were entered as continuous level data, and the remaining variables were entered as either dichotomous (sex, marital status, smoking status, place of screening, and health status) or categorical with dummy variables.

The main limitation of these data is that reported alcohol screening is based on recall from the time elapsed since the respondent’s last checkup. Additionally, because alcohol consumption is recorded for the time period proximal to the NHIS, our analysis assumes that this information is similar to consumption at the time of the physician visit.

To minimize these limitations, our analysis excludes respondents whose last checkup was longer than 2 years from the interview. This exclusion eliminated a greater proportion of men and younger respondents, because women and older respondents used physician services more frequently. To the extent that the excluded group may over- or underestimate the number of individuals screened for alcohol consumption by physicians, some biasing might be present.^5^ The benefit gained by including only those individuals with relatively recent checkups, however, was believed to outweigh this limitation.

## Sample Characteristics and Overall Findings

Table 1 presents data on the unweighted frequencies and weighted percentages for the population by selected demographic characteristics and by physician screening for alcohol consumption during the clinic visit. Results are based on 29,725 respondents, with a weighted distribution of 58 percent female and 42 percent male. The majority of respondents interviewed for the HPDP reported that they were not screened for alcohol consumption during their last checkup. The total percentage of respondents reporting that they were screened for alcohol consumption was 39 percent.

The rates of screening for alcohol consumption differed for various demographic subgroups. Comparison of the percentages of women and men who reported screening indicated that men were more likely to be screened for consumption (44 percent) compared with women (35 percent). Similarly, blacks were more likely to be screened (44 percent) than whites (38 percent). Respondents screened for alcohol were more likely to be younger and more highly educated.

Also presented in table 1 are health characteristics and alcohol consumption, which were determined by HPDP interview and by physician screening during checkup. Health characteristics include self-perceived health status and place of screening. A relationship was observed between self-perceived health status and incidence of screening for alcohol consumption. Screening was done most often among respondents who reported very good or good health status (40 percent), compared with respondents who reported fair or poor health status (33 percent).

The type of facility the respondents visited for checkups was associated with different rates of screening for alcohol consumption. The vast majority of respondents receiving a checkup did so at a doctor’s office (89 percent); however, this was the place where screening was least likely to occur. Clinics were reported as the facility where alcohol screening was most likely to occur (50 percent). The data indicated that 36 percent of respondents seen at a doctor’s office were screened for alcohol consumption, compared with 49 percent seen at a hospital outpatient facility, and 46 percent seen at other facilities.

Drinking status was compared with physician screening in the total population of respondents and separately for men and women (table 2). When physician screening was compared with drinking status within the total population of respondents, results indicated that abstainers were screened less frequently than all categories of current drinkers and that screening increases with increasing levels of consumption. Screening for alcohol consumption among current drinkers ranged from 38 percent for infrequent drinkers to 52 percent for heavy drinkers. Similar trends were observed when the population was separated by sex. Overall, women were screened less frequently than men; however, the pattern of screening by drinking status is the same for both sexes.

**Figure 1:**
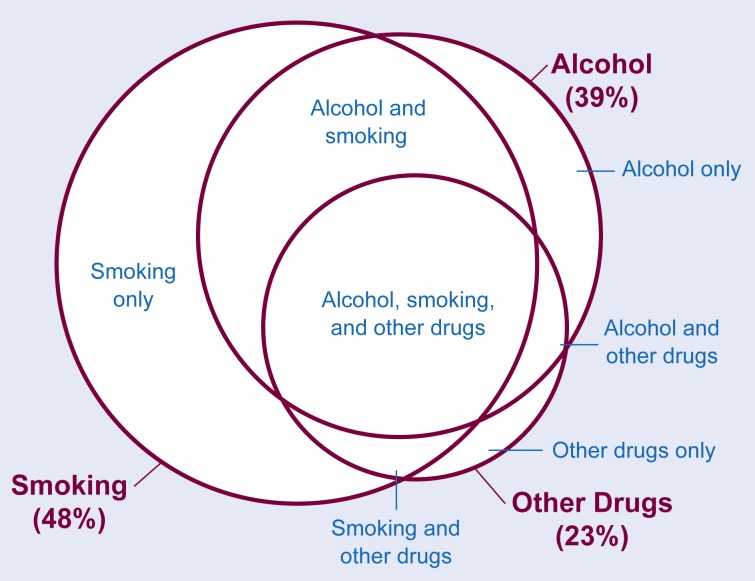
Interrelationships between screening for alcohol, smoking, and other drug use. The total percentages of persons reporting screening for alcohol, smoking, and other drugs are represented in the large circles and bold print. The overlap between screening for individual combinations of these variables is presented in the smaller spheres and print.

The percentages of respondents screened for smoking and drug use are presented in table 2. Screening for smoking occurred with a higher frequency than screening for alcohol and other drug use. Forty-eight percent of respondents were screened for smoking, 39 percent were screened for alcohol use (see table 1), and 23 percent were screened for other drug use. Among respondents screened for smoking, 72 percent also were screened for alcohol use; and among respondents screened for other drug use, 91 percent were screened for alcohol use. A higher percentage of current smokers were asked about their alcohol consumption compared with nonsmokers; however, 56 percent of current smokers were not asked about their alcohol consumption. The interrelationships between screening for alcohol, smoking, and other drug use are presented in figure 1.

Table 3 presents the results of the multiple logistic regression analysis. Only predictor variables that were statistically significant are included in the table. Variables that dropped out of the model included race, marital status, perceived health status, and poverty index. Variables with a significant association with screening are reported with their standard error and odds ratio. Both age and physician office were associated negatively with screening, indicating that older respondents were less likely to be screened, as were respondents visiting a private physician’s office compared with those visiting a hospital, clinic, or outpatient facility.

**Figure 2:**
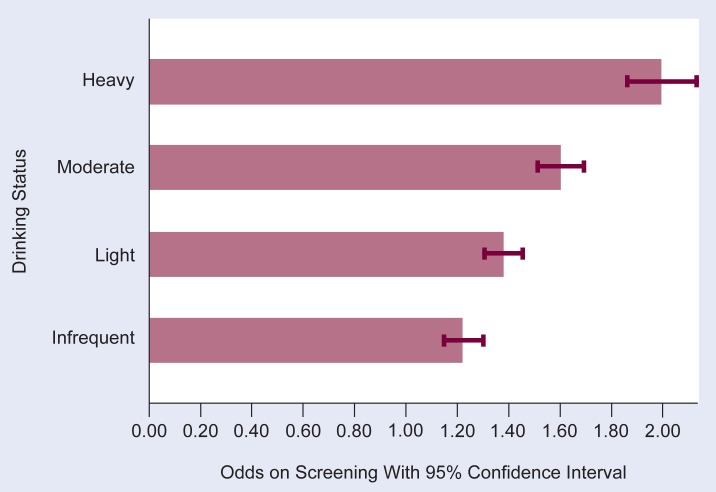
Odds ratios^1^ of physician screening by drinking status.^2^ NOTE: The reference group for the odds ratios is the abstainers. ^1^The term “odds ratio” and other terms are defined in the glossary (p. 163). ^2^Drinking status is defined as follows: abstainer = 0 drinks in past year; infrequent drinker = 0 drinks in past 2 weeks but 1+ drinks in past year; light drinker = 1–7 drinks in past 2 weeks; moderate drinker = 8–26 drinks in past 2 weeks; and heavy drinker = 27+ drinks in past 2 weeks.

Figure 2 presents the odds ratios of physician screening for the levels of alcohol consumption. The reference group for the odds ratios is the abstainers. Respondents reporting the highest alcohol consumption were most likely to have been screened, and all levels of current consumption were significantly more likely to have been screened when compared with abstainers.

## Discussion

Results of our analysis indicated demographic and health differences in the frequency of physician screening for alcohol consumption during office visits. A majority of respondents seen for a checkup within the past 2 years were not asked about their alcohol consumption. This finding has important implications for health promotion activities designed to meet year 2000 health promotion goals mentioned earlier. Namely, there should be increased involvement by primary care providers in discussing potentially harmful health behaviors with their patients, such as drinking. This is particularly important in light of the lack of a national effort to identify asymptomatic individuals with alcohol problems that are comparable in scope to national screening programs for diabetes, hypertension, high cholesterol, and cancer.

A report issued by The Institute of Medicine in 1990 included a recommendation for routine alcohol screening of all persons presenting themselves for care in medical settings, implementation and monitoring of a brief intervention if mild to moderate problems are found, and referral to specialized treatment for severe problems.

Numerous countries, including Great Britain, Canada, and Australia, have implemented screening for alcohol and brief intervention programs into their health care systems (Skinner 1990). The goal of brief intervention strategies is to prevent problems in individuals who are experiencing adverse effects of drinking but who are not physically dependent on alcohol (Babor and Grant 1989).

Compared with screening for other health behaviors, screening for alcohol consumption was found to occur less frequently than screening for smoking, but it occurred at a greater frequency than screening for use of other drugs such as marijuana and cocaine. Given the interrelationships between smoking and drinking, in which smokers are likely to be heavy drinkers and heavy drinkers are likely to smoke, screening for alcohol consumption should occur with at least the same frequency as screening for smoking (Bien and Burge 1990).

Important demographic and socioeconomic differences were observed between screened and nonscreened respondents. The findings that younger, more highly educated, and male respondents were screened most frequently have public health implications. Further studies are needed to obtain a more complete picture of the physician decisionmaking processes and aspects of doctor-patient interactions that determine when physician screening for alcohol consumption is most likely to occur.

The finding that screening for alcohol decreased as age increased is of particular interest. Although our analysis was not designed to investigate the potential reasons behind this finding, several statements about alcohol use and aging can be made. For example, longitudinal research on alcohol consumption indicates that drinking patterns appear to be relatively stable across time (Dufour et al. 1988).

Studies on life course variation in drinking indicate that drinking patterns are often erratic in late adolescence and early adulthood and tend to become increasingly stable with increasing age (Fillmore et al. 1991). Additionally, evidence from several studies indicate that there is a higher chronicity of heavy drinking in middle age; however, the overall prevalence of this behavior in midlife is lower than in the earlier adult years (Fillmore et al. 1991). Finally, though not common, an increase in drinking can occur later in life, and physicians should not assume that alcohol problems are unlikely to occur in an older patient.

Drinking in an elderly population can contribute to poorer health and may lead to more frequent visits to a physician. This finding may be related to work by Stinson and colleagues (1989), on the underdetection of alcohol-related morbidity in the elderly population. Results of the study indicated that there is considerable “hidden” alcohol-related morbidity among people age 65 and older.

The finding that the types of health care settings the respondents visited for checkups seemed to influence whether they were screened for alcohol consumption deserves further study. The increased reporting of alcohol screening in settings other than private offices may be related to particular procedures or policies at these facilities. Exploration of screening practices in health maintenance organizations (HMO’s), hospitals, and employee clinics compared with practices in private offices, where less screening is done, is worthy of future investigation to determine the reason for this finding.

An encouraging finding of this analysis is that screening for alcohol consumption appears to increase with increasing levels of drinking status. A larger percentage of respondents most at risk for health problems (i.e., the moderate to heavy drinkers) report that they were questioned about their alcohol consumption. Exploration of the processes by which physicians “knew” drinking levels of the patient deserves further attention. Possible explanations for this finding are the presence of clinical signs indicative of increased consumption or respondent bias or both in recollection of screening (e.g., heavier drinkers may be more cognizant of physicians’ questions regarding their drinking).

Less encouraging, however, is the finding that within the heavy consumption group, 48 percent report that they were not screened for alcohol consumption. Consequently, a substantial percentage of people with a known risk for health problems secondary to alcohol consumption are reporting a lack of questioning or a lack of involvement by their medical care provider or both. Additional efforts are needed within this population to help reduce alcohol-related health problems.

## Summary

These study findings allow us to develop several hypotheses regarding physicians’ practice of screening for alcohol consumption. Although these factors are not predictors of screening given the limitations of the study design, this work can be used as a basis for future studies investigating the relationships under consideration.

These findings point to a need for more research in the area and for increased awareness of the potential for underdetection of alcohol problems in a clinical setting. The clinic or office visit presents a valuable opportunity for detection and intervention aimed at preventing health problems and is worthy of increased efforts to direct these important activities. Physicians ought to be aware of the health risks posed by alcohol, the early warning signs of alcohol-related symptomatology, and the utility of asking patients about their alcohol consumption in addition to their smoking behavior.

Brief interventions for reducing alcohol use in the nondependent drinker should be incorporated into our health care system. These interventions typically are clinically based and inexpensive and include techniques such as assessment and direct feedback, the use of written materials such as self-help manuals, contracting and goal setting, and behavioral modification (Babor 1990).

Further research is needed to determine the most cost-effective screening procedures and to develop screening methods with improved reliability in detecting alcohol problems among women, minorities, and the elderly. Investigation of the effect of physician advice on alcohol use and brief interventions to reduce problem drinking is necessary for making recommendations on quality issues such as timeliness of intervention, appropriateness of the technique or procedure, and relevance of the technique/procedure to the recipient. Improved and more extensive research on doctor-patient interaction during office visits can serve to increase our understanding of health promotion activities and heighten awareness of the issues surrounding alcohol and health.

## Figures and Tables

**Table 1:** Selected Demographic Characteristics of Respondents Given in Total and by Screening for Alcohol Use by Physician During Checkup

	Unweighted Frequency	Weighted Percentage	Percentage Screened for Alcohol Consumption		

			Total	Male	Female
					
Total	29,725	100	39	—1	—
Sex					
Male	10,795	42	44	—	—
Female	18,930	58	35	—	—
Race					
White	23,925	83	38	43	33
Black	4,500	12	44	45	42
Other^2^	1,246	5	42	45	40
Age Group					
18–29 years	6,235	23	45	46	44
30–44 years	9,144	31	45	51	40
45–64 years	7,720	27	38	46	32
65+ years	6,626	19	22	27	18
Education (years in school)					
0–11 years	6,340	20	31	34	29
12 years	10,910	38	38	43	34
13–15 years	6,304	21	41	46	38
16+ years	6,115	21	45	51	39
Marital Status					
Currently married	16,224	65	39	44	34
Not currently married	13,473	35	61	44	36
Total Family Income					
Under $10,000/year	4,310	12	37	42	35
$10,000–$19,999/year	5,221	18	35	38	34
$20,000–$34,999/year	6,336	26	38	42	34
$35,000–$49,999/year	4,124	19	41	46	37
$50,000+/year	4,985	25	46	52	40
Poverty Index					
Currently in poverty	3,560	10	41	45	40
Not in poverty	26,165	90	38	44	35
Place Went for Checkup					
Doctor’s office	23,301	89	36	41	33
Hospital	1,727	6	49	51	47
Clinic	1,133	4	50	48	43
Other	241	1	46	48	46
Self-perceived Health Status					
Very good/good	25,691	86	40	45	36
Fair/poor	4,517	14	33	38	30

NOTE: Frequencies of classification variables may not sum to the total unweighted frequency because of missing values.

1 Dash indicates data not applicable.

2 Other races include Aleut, Eskimo, and American Indian; Asian/Pacific Islander; and multiple race.

**Table 2:** Health Characteristics and Self-Reported Drinking of Respondents Given in Total and by Screening for Alcohol Use by Physician During Checkup

	Unweighted Frequency	Weighted Percentage	Percentage Screened for Alcohol Consumption		

			Total	Male	Female
Drinking Status^1^					
Infrequent	6,317	22	38	35	28
Light	7,749	27	42	42	36
Moderate	3,712	13	48	45	40
Abstainer	10,193	33	30	51	43
Heavy	1,463	5	52	54	45
Smoking Status					
Current smoker	7,241	24	44	48	40
Not current smoker	22,777	76	37	42	33
Physician Screened for Smoking					
Yes	14,116	48	72	74	70
No	15,381	52	7	10	6
Physician Screened for Other Drug Use					
Yes	6,607	23	91	89	93
No	22,784	77	22	26	20

NOTE: Frequencies of classification variables may not sum to the total unweighted frequency because of missing values.

1 Drinking status is defined as follows: abstainer = 0 drinks in past year; infrequent drinker = 0 drinks in past 2 weeks but 1+ drinks in past year; light drinker = 1–7 drinks in past 2 weeks; moderate drinker = 8–26 drinks in past 2 weeks; and heavy drinker = 27+ drinks in past 2 weeks.

**Table 3:** Variables With a Significant Association With Screening as Determined by Results of Multiple Logistic Regression Analysis

Variable	Regression Coefficient	Standard Error	Odds Ratio
Intercept^1^	−0.499	0.144	0.607
Male	0.281	0.032	1.326
Private office	−0.300	0.038	0.741
Age	−0.015	0.000	0.985
Education	0.041	0.006	1.040
Current smoker	0.116	0.037	1.123
Infrequent drinker	0.194	0.044	1.215
Light drinker	0.321	0.042	1.379
Moderate drinker	0.473	0.052	1.604
Heavy drinker	0.684	0.073	1.982

NOTE: Age and education were entered as continuous level variables.

Reference groups for: males = females; private office = hospitals, clinics, and outpatient facilities; current smoker = not currently smoking; and the four levels of drinking = abstainers.

1 The term “intercept” and other terms are defined in the glossary (p. 163).

## Footnotes

1 Differences in the wording of alcohol questions on the 1991 HPDP preclude using similar or comparable classifications.

2 For a definition of this term and other terms used in this article, see the glossary on p. 163.

3 Data were weighted to reflect 1991 population estimates for sex, race, and age and to account for the selection of one adult per family and nonresponse.

4 This “worst case” effect was achieved by adjusting the weight variable provided with the 1991 NHIS data. The original weight variable was first divided by the full-sample mean weight to eliminate the statistical significance attributable to the increased size of the weighted sample. The new weight variable was then divided by 1.44 to increase the sample variance and, hence, the standard error by 20 percent, which is the “worst case” effect from the sampling design. A complete description of this methodology can be obtained from NCHS and is described, in part, in the technical appendixes of the *Current Estimates From the National Health Interview Survey, 1991* (U.S. Department of Health and Human Services 1992).

5 Biasing would be present if individuals seeing a physician within the last 2 years differ from those who did not see a physician in ways that may be related to screening.

## GLOSSARY

Confidence interval (CI): Consists of estimates located between two boundary points. The CI gives a specified level of confidence (e.g., 95%) that the estimate which falls within these parameters is not due to change.
Dummy variables: Variables with assigned values (e.g., 1 indicates the presence of a condition and 0 indicates the absence of the condition), which have been constructed to allow a comparison of categories within a given population.
Intercept: Point at which the sloping line meets the “Y” axis in a graph of a *regression analysis*. This line gives a graphic representation of the *regression analysis* equation.
Longitudinal study: Study of behaviors or conditions in the same population over at least two points in time.
Odds ratio: The ratio of probability that a factor will be present in two populations, each of which has different characteristics or exposures to a risk factor (e.g., the incidence rate of lung cancer for populations exposed to cigarette smoking and those who were not exposed).
Regression analysis: Analysis of the linear relationship (or association) of conditions to outcomes in a given population.
Regression coefficient: Measurement of the extent of the linear relationship between two variables. For example, if eating high fat foods is strongly related to being overweight, the regression coefficient would be high.
Sample variance: The amount of variation from the mean or average.
Standard error: An indicator of the variance (or dispersion) of an estimated value.
Unweighted numbers: Numbers that have not been manipulated or adjusted statistically to allow for sampling techniques.
Weighted numbers: Numbers that have been adjusted to allow for differences in sampling techniques. Weighting ensures that the proportion of the groups being studied is as close as possible to the actual proportion in the population.
